# Comparison of associations between alcohol consumption and metabolic syndrome according to three definitions: The Swedish INTERGENE study

**DOI:** 10.1016/j.metop.2024.100292

**Published:** 2024-06-05

**Authors:** Alina Skultecka, Fredrik Nyberg, Lauren Lissner, Maria Rosvall, Dag S. Thelle, Anna-Carin Olin, Kjell Torén, Lena Björck, Annika Rosengren, Kirsten Mehlig

**Affiliations:** a School of Public Health and Community Medicine, Institute of Medicine, University of Gothenburg, Gothenburg, Sweden; b Socialmedicinskt centrum, Regionhälsan, Västra Götalandsregionen, Sweden; c Department of Biostatistics, Institute of Basic Medical Sciences, University of Oslo, Norway; d Occupational and Environmental Medicine, School of Public Health and Community Medicine, Institute of Medicine, Sahlgrenska Academy, University of Gothenburg, Gothenburg, Sweden; e Department of Molecular and Clinical Medicine, Sahlgrenska Academy, University of Gothenburg, Gothenburg, Sweden; f Department of Medicine, Geriatrics and Emergency Medicine, Sahlgrenska University Hospital, Östra Hospital, Region Västra Götaland, Gothenburg, Sweden; g Sahlgrenska University Hospital/Östra, Region Västra Götaland, Gothenburg, Sweden

**Keywords:** Alcohol, Metabolic syndrome, Population-based study, Observational study, Confounding

## Abstract

**Background:**

While prevalence estimates differ by definition of metabolic syndrome (MetS), it is less clear how different definitions affect associations with alcohol consumption.

**Methods:**

We included 3051 adults aged 25–77 from the baseline examination of the Swedish INTERGENE cohort (2001–2004). Using multiple logistic regression, we investigated cross-sectional associations between ethanol intake and MetS defined according to the Adult Treatment Panel III (ATP III), the International Diabetes Federation (IDF), and the Joint Interim Statement (JIS). Alcohol exposure categories comprised abstinence, and low, medium, and high consumption defined via sex-specific tertiles of ethanol intake among current consumers. Covariates included sociodemographics, health, and lifestyle factors.

**Results:**

MetS prevalence estimates varied between 13.9 % (ATP III) and 25.3 % (JIS), with higher prevalence in men than women. Adjusted for age and sex, medium-high alcohol consumption was associated with lower odds of MetS compared to low consumption, while no difference was observed for abstainers. Only the most specific (and thus severe) definition of MetS (ATP III) showed decreasing odds for ethanol intake when adjusted for all covariates.

**Conclusion:**

Our study shows that alcohol-related associations differ by definition of MetS. The finding that individuals with the most stringently defined MetS may benefit from alcohol consumption calls for further well-controlled studies.

## Highlights

• We compare associations between alcohol consumption and MetS by 3 common definitions.
• High versus low ethanol intake is consistently associated with reduced odds for MetS.
• Confounders include diabetes heredity, sociodemographic, health & lifestyle factors.
• Only associations with MetS by ATP III remain after confounder adjustment.

## Graphical abstract

**Figure:**
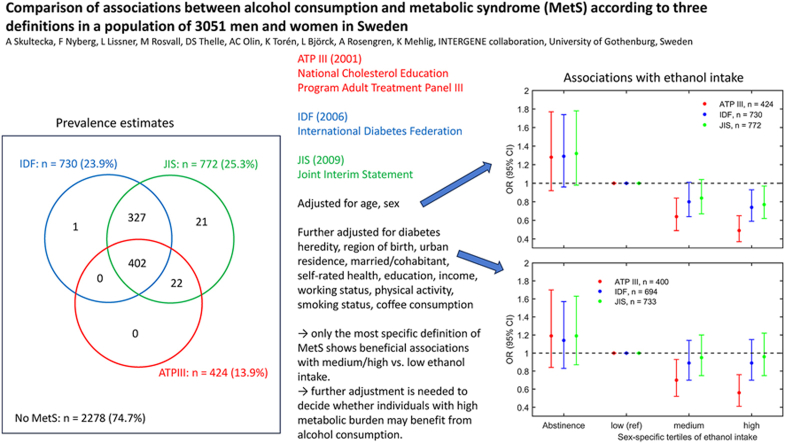

## 1 Introduction

The term Metabolic syndrome (MetS) describes the clustering of interrelated metabolic risk factors associated with atherosclerosis and cardiovascular disease [[1], [2], [3]]. Driven by the obesity epidemic, its prevalence increased to up to 40 % worldwide [4]. However, prevalence estimates differ strongly according to the cut-points used to define the individual conditions of MetS, i.e. large waist circumference, high blood pressure, high blood sugar level, high triglycerides, and low HDL cholesterol [[1], [2], [3]].

Age, obesity, and sedentary lifestyle are the main risk factors for MetS. Other risk factors include male sex, some ethnicities, low socioeconomic status, smoking, and abstention from alcohol consumption [5]. Protective associations between moderate alcohol consumption and health outcomes have been reported for coronary heart disease, dementia, and all-cause mortality [[6], [7], [8]], which may be related to increased HDL cholesterol and insulin sensitivity [9,10]. However, beneficial effects of moderate alcohol consumption on the cardiovascular system may not outweigh the risk of digestive disease and cancer [11,12] as well as alcohol abuse and addiction [13].

Using case-control data from the western-Swedish INTERGENE study we previously reported a U-shaped risk curve for alcohol consumption and coronary heart disease that was not confounded by other risk factors including HDL cholesterol [14]. A cohort study of women from the same region showed that any amount of alcohol was a risk factor for incident liver disease [15]. Given that MetS combines indicators of cardiovascular as well as metabolic disorder we now investigate the association between alcohol and MetS using baseline data from the well-characterized INTERGENE cohort. Bearing in mind the heterogeneity of MetS definitions, we use the three most recent definitions, i.e. from the National Cholesterol Education Program Adult Treatment Panel III (ATP III) [16], the International Diabetes Federation (IDF) [17] and the Joint Interim Statement (JIS) [18]. We aimed to answer three research questions: What are the prevalence estimates for MetS in western Sweden in 2001–2004? Are there associations with habitual alcohol consumption and how do they differ by definition of MetS? To which extent are potential associations reduced by confounders accounting for individual differences in health, socioeconomic status, and lifestyle?

## 2 Material and methods

### 2.1 Study population

The INTERGENE research programme was initiated to study the INTERplay between GENEtic and environmental risk factors for chronic disease. A population-based cohort of 3614 adult individuals, aged 25 to 77, living in Västra Götaland County, Sweden, was examined in 2001–2004. About half of them lived in Gothenburg (the second-largest city in Sweden) and 88 % were born in Sweden. The participation rate was 42 % [19]. The analytic sample in this study included only participants who had complete data on MetS and alcohol consumption (n = 3051).

### 2.2 Data collection methods

Lifestyle and sociodemographic characteristics as well as use of medications were obtained through self-administrated questionnaires. Anthropometric characteristics and blood samples were obtained at physical examinations, which took place at the Sahlgrenska University Hospital in Gothenburg or in a mobile research bus for participants who lived in rural areas [19].

### 2.3 Anthropometry and blood biomarkers

Waist circumference was measured midway between the lower rib margin and the iliac crest. Height and weight were measured to the nearest cm and 0.1 kg, respectively, with participants in light clothing without shoes. Body mass index (BMI) was calculated as weight/height^2^ and categories were defined using cut-points given by 20, 25, 30, and 35 kg/m^2^. Blood pressure was measured twice after 2 min rest using an inflationary oscillometric blood pressure apparatus (Omron 711 Automatic IS), and the mean value was calculated. Venous blood samples were collected, after a minimum of 4-h fasting, into tubes containing 0.1 % EDTA for immediate laboratory analysis. The concentration of serum triglyceride was determined using enzymatic assays. The concentration of serum HDL cholesterol concentrations was measured by dextran sulphate-magnesium precipitation of apoB-containing lipoproteins. The Hexokinase method (Roche Hitachi 917 and Roche ModularP, Roche, Basel, Switzerland) was used to estimate plasma glucose [19].

### 2.4 Definition of metabolic syndrome

We used three definitions for metabolic syndrome, from the National Cholesterol Education Program Adult Treatment Panel III (ATP III) [16], the International Diabetes Federation (IDF) [17] and the Joint Interim Statement (JIS) [18]. All three definitions have the same components but the ATP III definition has higher cut-points for fasting plasma glucose and waist circumference than the IDF and JIS definitions, and the IDF definition includes central obesity as a mandatory criterion (Table 1). The ATP III definition was included to be able to compare the prevalence of MetS with previous Swedish studies, while the most recent definition (JIS) was given for future reference. The IDF definition was selected because it is most widely used nowadays.

**Table 1:** Criteria for clinical diagnosis of metabolic syndrome by organisation.

	ATP III (2001) [16]	IDF (2006) [17]	JIS (2009) [18]
**Diagnosis criteria**	Any 3 of 5 criteria	Central obesity and ≥2 other criteria	Any 3 of 5 criteria
**Central obesity**	Waist circumference >102 cm (men) >88 cm (women)	Waist circumference ≥94 cm (men)^b^ ≥80 cm (women) or BMI >30 kg/m^2^	Waist circumference ≥94 cm (men)^b^ ≥80 cm (women)
**Elevated fasting glucose**	≥6.1 mmol/l	≥5.6 mmol/l or type 2 diabetes	≥5.6 mmol/l or medication
**Hypertension**	≥130/85 mmHg or medication	≥130/85 mmHg or medication	≥130/85 mmHg or medication
**Elevated triglycerides**	≥1.7 mmol/L	≥1.7 mmol/L or medication^c^	≥1.7 mmol/L or medication^c^
**Reduced HDL cholesterol**^a^	<1.03 mmol/L (men) <1.29 mmol/L (women)	<1.03 mmol/L (men) <1.29 mmol/L (women) or medication^c^	<1.03 mmol/L (men) <1.29 mmol/L (women) or medication^c^

National Cholesterol Education Program Adult Treatment Panel III (ATP III).

International Diabetes Federation (IDF).

Joint Interim Statement (JIS).

a Cut-points in mmol/L corresponding to 40 mg/dL and 50 mg/dL for men and women, respectively.

b a lower cut-point of 90 cm was used for men born in Asia and Central/South America (n = 14).

c Due to lack of information on lipid-specific medication this criterion was not used in the definition of elevated triglycerides or reduced HDL cholesterol in this study.

### 2.5 Alcohol consumption

The questions about the consumption frequency of alcoholic beverages were included in a validated food frequency questionnaire assessing dietary habits during the last year [14,19]. Participants reported their habitual consumption of six types of alcoholic beverages (approximate volume percent alcohol given in parentheses): light beer class I (3 %), light beer class II (4 %), beer (6 %), wine (11 %), fortified wine (30 %) and spirits (41 %). The total daily ethanol intake was calculated as the product of standard beverage portion size (cl), ethanol content (g/cl) and consumption frequency (portions/day). Because the INTERGENE questionnaire did not include questions on portion size we used previously developed age- and sex-specific standard portion sizes [14]. Daily ethanol intake in grams (g/day) was categorized into “Abstinence from alcohol” and three categories of current use based on sex-specific tertiles, “Low alcohol consumption” (<6.9 g/day for men and <3.1 g/day for women), “Medium alcohol consumption” (6.9–13.4 g/day for men and 3.1–6.5 g/day for women), and “High alcohol consumption” (>13.4 g/day for men and >6.5 g/day for women). A posteriori analyses aimed to assess whether alcohol-related associations with metabolic outcomes differed by subtype of alcoholic beverage. To this end, we calculated the ethanol intake from beer (combining the three subtypes with differing alcohol percentage), wine (combined with strong wine) and spirits, which were categorized using the same cut-points as for total ethanol intake. This approach allowed to compare the metabolic associations of individual beverage types with approximately the same ethanol content in mutually adjusted regression models.

### 2.6 Potential confounders

A number of variables associated with alcohol consumption and metabolic health were selected among those measured in INTERGENE. The basic model for MetS included sex and age, whereby age was recoded into a categorical variable with 10-year intervals to allow for non-linear associations. Country of birth was used as a proxy for ethnicity, which is related to use of alcohol as well as metabolic disorder [5]. Because the majority of participants were born in Sweden, Norway or Finland we only distinguished between born in Nordic countries or not. Heredity of diabetes was included as a risk for MetS and was derived from reports of parental disorder (any parent vs. none). Health problems may be an important reason for which individuals decide to restrict their alcohol consumption. To account for this aspect we adjusted for self-rated health with response categories very good, good, mixed, and poor, whereby the two latter were combined. Self-rated health has been shown to be a better predictor of health outcomes than medical assessment [20] and accounts for the individuals own perception, which in turn determines their lifestyle choices. Social factors include being married/cohabitant versus living alone, and living in a town (Gothenburg) vs. countryside. Self-reported education was dichotomized into university education versus less education. Information on working status was categorized as follows, working full/part-time, pensioner/early retirement/sick leave and unemployment/other. Information on income was lacking, but a variable for economic problems combined the answers to two questions, i.e. whether participants had economic problems in the last year and whether they could raise 14000 SEK (equivalent to 1600 EUR in 2024) within a week in case of an unforeseen emergency. If participants had economic problems or could not raise 14000 SEK, they were assigned low-income status (yes/no). Leisure time physical activity was recorded into three groups: sedentary behaviour, moderate physical activity (at least 4 h per week) and regular training or competitive sports. Smoking was divided into never, former, and current smoking. Coffee consumption categories were none, under 3 cups/day, and ≥3 cups/day, where cut-off points were chosen to obtain categories of similar size.

### 2.7 Statistical methods

The prevalence of MetS according to the three definitions was estimated with 95 % confidence intervals (CI). Agreement between the different definitions was assessed using the Cohen’s kappa-coefficient. Chi-square tests were used to compare categorical variables across categories of ethanol intake. The associations between alcohol consumption and MetS as well as its individual components according to three definitions were investigated using multiple logistic regression. The reference category for alcohol exposure was low ethanol intake, because abstainers are generally a heterogeneous group including teetotalers as well as former users and abusers of alcohol, with rather different health status [21]. Effect modification by sex was tested by including product terms for sex and ethanol categories in the regression models. The overall p-value for interaction was calculated by a likelihood-ratio test comparing a model without interaction terms with a model with all interaction terms. Results were given as odds ratios (OR) with 95 % confidence intervals (CI). Model fit was assessed in terms of the area under the ROC curve (AUROC). A posteriori analyses assessed the associations with ethanol intake form beer, wine, and spirits categorized using the same cut-points as for total ethanol intake in mutually adjusted regression models for MetS (ATP III) and low HDL cholesterol. Results with p-values <0.05 were considered statistically significant. Statistical analysis was performed using STATA v. 17.0 (StataCorp, Texas, USA).

### Ethical approval

The study was conducted in agreement with the declaration of Helsinki. All participants gave their written informed consent to the study. The protocol was approved by the regional ethics review board (reg no. Ö 237/2000).

## 3 Results

### 3.1 Prevalence of metabolic syndrome

The prevalence of MetS was highest by the most recent definition, 25.3 % (JIS), and smallest for the oldest definition, 13.9 % (ATP III), while the prevalence by the IDF definition was given by 23.9 % (Table 2). The prevalence of MetS was higher among men than women for all three definitions. Overall, 773 (25.3 %) of all participants had a diagnosis of MetS by at least one definition (Fig. S1). Among these, 402 participants (52 %) had a diagnosis according to all three definitions. The definition of MetS according to JIS is the most comprehensive (Table 1), including all other diagnoses with the exception of individuals without abdominal adiposity but with BMI >30 as well as ≥ two further criteria. In our sample, only one individual fulfilled these criteria (Fig. S1). Despite similar cut-off values, the prevalence of MetS according to the IDF definition was smaller than for JIS as the IDF definition has the additional requirement of central obesity. Cohen’s kappa indicated higher agreement between MetS based on the IDF and JIS definition, κ = 0.96, than between IDF and ATPIII, κ = 0.63, and between JIS and ATPIII, κ = 0.65, as also reflected in the greater similarity of prevalence estimates for the IDF and JIS definitions.

**Table 2:** Metabolic syndrome prevalence with 95% CI, overall and for men and women separately.

**MetS definition**	Male (n = 1453)		Female (n = 1598)			Total (n = 3051)	
	n	%	n	%	p-value	n	%
ATP III (2001)	225	15.5 (13.7–17.4)	199	12.5 (10.9–14.2)	*0.02*	424	13.9 (12.7–15.2)
IDF (2006)	423	29.1 (26.8–31.5)	307	19.2 (17.3–21.2)	*<*0*.0001*	730	23.9 (22.4–25.5)
JIS (2009)	454	31.2 (28.9–33.7)	318	19.9 (18.0–21.9)	*<*0*.0001*	772	25.3 (23.8–26.9)

Metabolic Syndrome (MetS).

National Cholesterol Education Program Adult Treatment Panel III (ATP III).

International Diabetes Federation (IDF).

Joint Interim Statement (JIS).

### 3.2 Characteristics of study participants and their association with ethanol intake

The mean age of participants was 51.5 years and 52 % of them were women. About 90 % of study participants reported current alcohol consumption. There were large differences in demographic, socioeconomic, health and lifestyle factors between the four categories of alcohol consumption (Table 3). Current abstainers were older than current consumers of alcohol, 55 vs. 51 years of age, and more likely to be women. Regarding trends across all four categories of ethanol intake, participants with higher ethanol intake were more likely to be born in a Nordic country, to live together with a partner, to score higher on self-rated health, to have no parental history of diabetes, to be working, and to not have economic problems compared to those with lower ethanol intake. Participants with medium and high ethanol intake were more likely to have university education than those with no or low ethanol intake. They were more physically active and reported higher coffee consumption. The group of current abstainers showed the largest percentage of never and of current smokers, while the proportion of former smokers was largest among those with high alcohol consumption. Mean BMI was highest among current abstainers (27.0) and decreased across ethanol tertiles according to 26.5, 25.9, and 25.2 (p < 0.0001).

**Table 3:** Characteristics of study participants from the INTERGENE study in western Sweden 2001–2004 by category of ethanol intake.

	Categories of current ethanol intake^b^			
	Abstinence (n = 311)	Low (n = 909)	Medium (n = 916)	High (n = 915)
***Ethanol intake (g/day)***^a^				
*Men*	0	0.8-6.9	6.9–13.4	>13.4
*Women*	0	0.4-3.1	3.1–6.5	>6.5
	n (%)	n (%)	n (%)	n (%)
***Demographic characteristics***				
*Female sex*	212 (68.2)	462 (50.8)	461 (50.3)	463 (50.6)
*Nordic born* *(n = 3036)*	253 (81.9)	809 (89.6)	848 (93.1)	859 (94.1)
*Age*				
25–34 years	35 (11.2)	154 (16.9)	115 (12.6)	82 (9.0)
35–44 years	50 (16.1)	189 (20.8)	200 (21.8)	209 (22.8)
45–54 years	50 (16.1)	166 (18.3)	212 (23.1)	228 (24.9)
55–64 years	80 (25.7)	210 (23.1)	218 (23.8)	238 (26.0)
65–77 years	96 (30.9)	190 (20.9)	171 (18.7)	158 (17.3)
*Married/cohabitant* *(n = 3036)*	194 (63.6)	652 (72.1)	708 (77.6)	756 (82.7)
*Urban residence*	167 (53.7)	454 (49.9)	455 (49.7)	512 (56.0)
***Health parameters***				
*Self-rated health* *(n = 3017)*				
Very good	38 (12.3)	147 (16.4)	202 (22.3)	233 (25.8)
Good	138 (44.8)	498 (55.4)	510 (56.3)	520 (57.5)
Mixed/poor	132 (42.9)	254 (28.3)	194 (21.4)	151 (16.7)
*Heredity for diabetes*	63 (20.3)	149 (16.4)	144 (15.7)	119 (13.0)
***Socioeconomic characteristics***				
*University education* *(n = 3034)*	78 (25.4)	218 (24.1)	292 (32.0)	342 (37.6)
*Low income* *(n = 3008)*	86 (28.0)	204 (22.6)	121 (13.3)	106 (11.6)
*Working status* *(n = 3037)*				
Working full/part-time	134 (43.8)	541 (59.7)	599 (65.6)	626 (68.6)
Retired	143 (46.7)	276 (30.5)	246 (26.9)	218 (23.9)
Unemployed, other	29 (9.5)	89 (9.8)	68 (7.5)	68 (7.5)
***Lifestyle factors***				
*Physical activity* *(n = 3041)*				
Sedentary	45 (14.7)	96 (10.6)	80 (8.8)	79 (8.7)
Moderate	210 (68.4)	575 (63.3)	569 (62.3)	565 (62.0)
Regular training	52 (16.9)	237 (26.1)	265 (29.0)	268 (29.4)
*Smoking* *(n = 3029)*				
Never	173 (55.8)	478 (53.1)	448 (49.0)	411 (45.4)
Former	71 (22.9)	265 (29.4)	314 (34.4)	351 (38.8)
Current	66 (21.3)	157 (17.4)	152 (16.6)	143 (15.8)
*Coffee consumption* *(cups/day, n = 3024)*				
None	63 (20.7)	97 (10.8)	73 (8.0)	42 (4.6)
< 3 cups/day	105 (34.5)	316 (35.0)	332 (36.5)	362 (39.8)
≥ 3 cups/day	136 (44.7)	489 (54.2)	504 (55.5)	505 (55.6)

a Range of values.

b Chi-square test for variation across categories of ethanol intake: heredity for diabetes (p = 0.02), urban residence (p = 0.02), physical activity (p = 0.0002), all other variables (p < 0.0001).

### 3.3 The association between alcohol consumption and metabolic syndrome

Associations between ethanol categories and metabolic syndrome according to all three definitions were not modified by sex or age (not shown), and subsequent association analyses were performed in the entire data set. Table 4 shows the odds ratio of having MetS and its components by category of ethanol intake adjusted for sex and age. High compared to low ethanol intake was associated with lower risk for MetS according to all three definitions but only MetS by ATP III definition showed a contrast between medium and low ethanol intake. A similar pattern was observed for the three definitions of central obesity. High vs. low ethanol intake was negatively associated with hypertension and elevated triglycerides. Low HDL cholesterol was the only outcome that showed a dose-response relationship with respect to ethanol intake, with a significant contrast between high and medium ethanol intake (p = 0.0004). No differences were observed between abstainers and consumers of alcohol except for triglycerides that were higher among current abstainers.

**Table 4:** Associations between alcohol consumption and metabolic syndrome and its components according to three definitions (adjusted for age and sex, n = 3051).

		Ethanol intake (ref = low)			
		Abstinence	Medium	High	
	Number of cases	OR (95 % CI)	OR (95 % CI)	OR (95 % CI)	AUROC (95 % CI)
MetS – ATP III	424	1.28 (0.92, 1.77)	0.64** (0.49, 0.84)	0.49*** (0.37, 0.65)	0.68 (0.66, 0.71)
MetS – IDF	730	1.29 (0.96, 1.74)	0.80 (0.64, 1.01)	0.74** (0.59, 0.93)	0.70 (0.68, 0.72)
MetS – JIS	772	1.32 (0.98, 1.78)	0.84 (0.67, 1.04)	0.77* (0.62, 0.97)	0.70 (0.68, 0.72)
Central obesity – ATP III	699	1.14 (0.86, 1.53)	0.67*** (0.53, 0.84)	0.60*** (0.48, 0.75)	0.67 (0.65, 0.69)
Central obesity – IDF	1646	1.13 (0.85, 1.49)	0.91 (0.75, 1.11)	0.74** (0.61, 0.90)	0.67 (0.65, 0.69)
Central obesity – JIS	1645	1.14 (0.86, 1.50)	0.92 (0.76, 1.11)	0.75** (0.61, 0.90)	0.67 (0.65, 0.68)
Elevated glucose – ATP III	230	0.69 (0.41, 1.18)	0.72 (0.50, 1.03)	0.89 (0.63, 1.25)	0.73 (0.70, 0.77)
Elevated glucose – IDF	576	0.80 (0.56, 1.14)	0.76* (0.59–**0.98)**	1.14 (0.89–1.45)	0.72 (0.70, 0.75)
Elevated glucose – JIS	564	0.82 (0.57, 1.18)	0.79 (0.61, 1.02)	1.15 (0.91, 1.47)	0.72 (0.70, 0.75)
Hypertension	1779	0.97 (0.71, 1.32)	0.85 (0.68, 1.05)	0.76* (0.61, 0.94)	0.78 (0.76, 0.80)
Elevated triglycerides	787	1.46** (1.10, 1.94)	0.83 (0.67, 1.03)	0.67*** (0.53, 0.83)	0.65 (0.63, 0.67)
Low HDL cholesterol	317	1.26 (0.88, 1.80)	0.69* (0.52, 0.93)	0.36*** (0.26, 0.51)	0.62 (0.58, 0.65)

Metabolic Syndrome (MetS).

National Cholesterol Education Program Adult Treatment Panel III (ATP III).

International Diabetes Federation (IDF).

Joint Interim Statement (JIS).

Area under the receiver operating characteristic curve (AUROC).

*p-value <0.05, ** p-value <0.01, *** p-value <0.001.

Associations between ethanol intake and MetS and central obesity remained only for outcomes according to the ATP III definition when further adjusted for additional confounders (Table 5). Also, high vs. low ethanol intake was associated with reduced odds for high triglycerides and low HDL cholesterol, with a significant difference between high and medium ethanol intake for the latter (p = 0.001).

**Table 5:** Associations between alcohol consumption and metabolic syndrome and its components according to three definitions and adjusted for all covariates in Table 3 (n = 2912)^a^.

Outcome		Ethanol intake (ref = low)			
		Abstinence	Medium	High	
	Number of cases	OR (95 % CI)	OR (95 % CI)	OR (95 % CI)	AUROC (95 % CI)
MetS – ATP III	400	1.19 (0.84, 1.70)	0.70* (0.52, 0.93)	0.56*** (0.41, 0.76)	0.76 (0.73, 0.78)
MetS – IDF	694	1.14 (0.83, 1.57)	0.89 (0.70, 1.14)	0.89 (0.70, 1.15)	0.74 (0.72, 0.76)
MetS – JIS	733	1.19 (0.87, 1.63)	0.95 (0.75, 1.20)	0.96 (0.75, 1.22)	0.74 (0.72, 0.76)
Central obesity – ATP III	657	1.09 (0.79, 1.50)	0.74* (0.58, 0.95)	0.67** (0.52, 0.86)	0.75 (0.73, 0.77)
Central obesity – IDF	1561	1.01 (0.75, 1.37)	1.02 (0.83, 1.26)	0.88 (0.71, 1.08)	0.71 (0.69, 0.73)
Central obesity – JIS	1560	1.02 (0.75, 1.38)	1.03 (0.84, 1.26)	0.88 (0.72, 1.09)	0.71 (0.69, 0.73)
Elevated glucose – ATP III	214	0.69 (0.39, 1.20)	0.77 (0.53, 1.14)	1.03 (0.71, 1.50)	0.79 (0.76, 0.82)
Elevated glucose – IDF	542	0.77 (0.53, 1.12)	0.80 (0.61, 1.05)	1.23 (0.95, 1.60)	0.75 (0.73, 0.77)
Elevated glucose – JIS	532	0.80 (0.54, 1.17)	0.83 (0.63, 1.09)	1.26 (0.97, 1.64)	0.75 (0.73, 0.77)
Hypertension	1695	1.08 (0.77, 1.51)	0.88 (0.70, 1.10)	0.88 (0.70, 1.11)	0.80 (0.78, 0.81)
Elevated triglycerides	745	1.27 (0.93, 1.72)	0.91 (0.72, 1.14)	0.77* (0.61, 0.97)	0.70 (0.68, 0.73)
Low HDL cholesterol	300	1.07 (0.73, 1.57)	0.76 (0.56, 1.02)	0.40*** (0.28, 0.58)	0.69 (0.66, 0.72)

Metabolic Syndrome (MetS).

National Cholesterol Education Program Adult Treatment Panel III (ATP III).

International Diabetes Federation (IDF).

Joint Interim Statement (JIS).

Area under the receiver operating characteristic curve (AUROC).

*p-value <0.05, ** p-value <0.01, *** p-value <0.001.

a Adjusted for sex, age, heredity for diabetes, born in Nordic countries, urban residence, married/cohabitant, self-rated health, education, income, working status, physical activity, smoking status, coffee consumption.

Table S1 shows the associations between individual covariates and metabolic endpoints according to ATP III. The ATP III definition was chosen as the corresponding models showed the highest values for the area under the ROC curve (Table 5). Higher age and male sex were associated with MetS and all its components except low HDL cholesterol, but women were at higher risk for central obesity according to all three definitions. To be born in a Nordic country was a risk factor for central obesity by ATPIII definition only. Parental history for diabetes was positively associated for MetS, central obesity, elevated glucose, and with low HDL cholesterol. Lower self-rated health was a strong positive predictor for all outcomes except low HDL cholesterol. Participants living in a town were less likely to have hypertension and more likely to have elevated glucose than those living in the countryside. Higher education or income as well as working status were associated lower risk for several components but not MetS itself. Former smoking was a risk factor for MetS and central obesity, while current smoking was associated with lower odds for hypertension and elevated glucose. Higher physical activity was inversely associated with MetS and its components except for hypertension and elevated glucose. Coffee consumption was weakly associated with more central obesity.

### 3.4 Sensitivity analyses

Further adjustment for BMI effaced ethanol-related associations with high triglycerides (not shown), but reduced associations with MetS by ATP III remained, OR = 0.74 (0.53, 1.01) and 0.67 (0.48, 0.95) for medium and high compared to low ethanol intake, respectively. Inverse associations between ethanol intake and risk for low HDL cholesterol were hardly affected by further adjustment for BMI, with corresponding ORs given by 0.78 (0.57, 1.06) and 0.44 (0.30, 0.64). Use of statins was associated with increased odds of low HDL cholesterol, OR = 1.75 (1.11, 2.77), but adjustment for statin use did not change the associations with ethanol intake (not shown). To be born in a Nordic country did not modify the association between alcohol and metabolic outcomes (not shown).

### 3.5 A posteriori analyses of subtypes of alcoholic beverages

To clarify the role of subtypes of alcoholic beverages we distinguished between ethanol intake from beer, combining the three subtypes of different volume percent, from wine or strong wine, and from spirits. For each participant, we calculated the percentage of ethanol intake due to beer, wine, and spirits, and compared the mean values by ethanol category among current consumers (Table S2). Wine was the most often consumed beverage type followed by beer and spirits, both overall and across categories of increasing ethanol intake.

To allow the comparison of associations with metabolic outcomes, ethanol intake from beer, wine, and spirits was categorized using the same cut-points as for total ethanol intake (section 2.5). Table S3 gives the association results for MetS – ATP III and for low HDL cholesterol in relation to ethanol intake from beer, wine, and spirits in mutually adjusted models. Consumption of both beer and wine was associated with lower odds for MetS and low HDL cholesterol but only associations with wine were significant. In contrast to models with total ethanol intake (Table 4), the age- and sex-adjusted models showed that abstinence from beer and wine were associated with increased odds for low HDL cholesterol or MetS, respectively, but the excess risk was no longer observed when adjusted for socioeconomic status and poor self-reported health. High ethanol intake from spirits was associated with higher odds for both outcomes, though significantly so only for low HDL-cholesterol in the age- and sex-adjusted model.

## 4 Discussion

The prevalence of MetS among the participants living in Västra Götaland County in 2001–2004 ranged from 13.9 % to 25.3 % depending on the MetS definition, with higher values for the more recent definitions of MetS. The prevalence estimates were consistently higher for men than for women. High and medium ethanol intake were associated with lower odds for MetS and central obesity by ATP III definition, and with low HDL cholesterol in age- and sex-adjusted models. Compared to ATP III, weaker associations with MetS and central obesity according to JIS/IDF definitions were observed in age and sex-adjusted models, but these were no longer statistically significant when adjustments were made in the full model. Current abstinence from alcohol was associated with higher odds for high triglyceride level in the age- and sex-adjusted model but not when further adjusted for confounding variables. A beneficial association between high ethanol intake and low triglycerides was explained by further adjustment for BMI. Although the covariates included in this study covered rather different areas of health and lifestyle they could not reduce the beneficial associations between ethanol intake and low HDL cholesterol as well as MetS and central obesity according to the ATP III definition. A posteriori analyses of subtypes of alcoholic beverages showed that the beneficial associations were due to consumption of wine and beer, while spirits consumption showed adverse associations with metabolic outcomes.

As expected, the prevalence of MetS was higher for the more recent MetS definitions that used lower cut-off points for central obesity and elevated glucose. Our estimates are consistent with results from a contemporaneous study of middle-aged men and women in Eastern Sweden (Linköping, 2003–04) that obtained a prevalence of 15 % according to ATP III [22]. While prevalence estimates according to IDF were comparable with those from an earlier study in Southern Sweden (Malmö, 1991–94) [23], their estimates of MetS by ATP III were somewhat higher (21 %) than in our study (14 %), possibly indicating that population-specific differences including non-participation are more important for the more specific than for the more comprehensive outcome definitions.

Our study shows that the risk for MetS among current abstainers was comparable to the risk among participants with low alcohol consumption, and that the risk decreased with higher ethanol intake among current consumers. The excess risk for elevated triglycerides among abstainers was reduced when adjusted for confounders including BMI, low self-rated health, and indicators of low socioeconomic status and unhealthy lifestyle, which were most prevalent among abstainers. Confounding with poor health status was convincingly demonstrated to explain the excess risk at the low end of the U-shaped risk curve for alcohol and mortality or CVD [21,24] and was the reason for using the category of low ethanol intake as reference in the present study. Compared to low intake, the risk reduction associated with medium and high ethanol intake was observed only for the ATP III definition of MetS, which is the most specific one of all three definitions, due to higher cut-points for central obesity and glucose. This difference is illustrated by higher prediction accuracy for the ATP III definitions of MetS, central obesity, and glucose measured in terms of AUROC. The Malmö study cited above showed that MetS by the ATP III definition was a better predictor of incident CVD than MetS by IDF [23]. In turn, MetS by the WHO definition (not used in our study) was found to give even higher risk estimates of CVD than the ATP III definition, which was ascribed to the fact that the WHO definition describes a more severe condition compared to the later ATP III definition [25]. Taken together, the results suggest that individuals with the highest metabolic burden (in our study represented by MetS - ATP III) and risk of CVD may profit the most from beneficial risk factors including alcohol consumption. However, the observation that BMI reduces the beneficial associations with MetS suggests that there is residual confounding by other factors responsible for the better health of alcohol consumers compared to those with low or no alcohol consumption. In contrast, low HDL cholesterol was the only component of MetS consistently showing a reduced risk for higher ethanol intake that was hardly affected by further adjustment for BMI or statin use. This finding is in agreement with results from previous studies and supports the hypothesis of a genuine biological mechanism [9,14,26]. A posteriori analyses showed that the beneficial associations with HDL cholesterol were mostly due to wine and beer consumption, suggesting a role for compounds other than ethanol, such as antioxidants. In contrast to adverse associations with ethanol from spirits, the associations with wine were also observed after adjustment for confounders, and the independence of living conditions further supports a direct role of wine or beer. Our results that wine and beer have opposite associations with MetS and HDL cholesterol compared to spirits are consistent with study results from China [27] and Brazil [26], and with results from a white population in the US reporting beneficial associations between consumption of beer and wine and MetS by ATP III definition, and no association with exclusive consumption of spirits [28]. Finally, the monotonic decrease in risk of MetS may appear to be in conflict with results from other studies showing excess risk at the high end of ethanol intake, however, this risk was observed at considerably higher levels of ethanol intake than reported in the INTERGENE population [29,30].

### 4.1 Strengths and limitations

The strength of the present study is the large sample size and standardised measurement of anthropometric characteristics and blood biomarkers. However, there was a selection bias in the INTERGENE study because non-participation was higher among economically deprived adults [31]. Because socioeconomic factors were associated with MetS, this bias may have caused an underestimation of the prevalence estimates for MetS in this study. Alcohol-related associations, on the other hand, may be less affected by non-participation as regression models included detailed information on education and socioeconomic status. Alcohol consumption was self-reported, which may have led to underreporting due to perceived stigmatisation if truthfully reported [32], but the placement of alcohol-related questions in a food frequency questionnaire may be an advantage [33]. The cross-sectional nature of the study is a limitation, as alcohol consumption may have changed due to prevalent health problems, which hampers the interpretation of the former as a risk factor for MetS. Also, the fact that the data for the study was collected two decades ago is a limitation. Last, residual confounding with e.g. ethnicity or genetics of ethanol metabolism is an important limitation that might be responsible for the beneficial associations between alcohol and MetS observed in this mostly Swedish population. Previous research has shown that alcohol metabolism as well as consumption patterns vary between different ethnic groups [34], and by genotypes within ethnicities [35,36], which the country of birth assessed here cannot account for.

### 4.2 Conclusions

Our study is one of few studies of alcohol-related associations with MetS in a predominantly Caucasian population [29], and it is the first one to compare alcohol-related risk with respect to three common definitions of MetS. While adjustment for various health-related risk factors reduced the inverse associations with the more comprehensive IDF and JIS definitions to non-significance, the association with MetS according to ATP III remained. In contrast to HDL-cholesterol, where causal effects of alcohol are plausible, results for MetS are still inconclusive, for instance as they lack the clear dose-response relationship observed for HDL-cholesterol. Future studies should include further risk factors related to the consumption and metabolism of alcohol including genetic and dietary factors [37]. The observation that not only prevalence estimates but also association results differ by MetS definition will be of interest for both epidemiologists and policy makers.

## Availability of data and materials

The data are available upon request and approval by the scientific review board.

## Funding

The study was funded by the Swedish Research Council for Working Life and Social Research (FORTE) (2001–0263, 2003–0139, 2006-1506, 2018–02527), the Swedish Research Council (VR) (2008–4015, 521-2010-2984), the Swedish Heart and Lung Foundation (2005-0561, 2009-0390, 2021-0345), the Swedish Research Council for Environment and Spatial Planning (FORMAS) (222-2006-1242, 216-2008-1203, 2021-493), the Västra Götaland County Council (ALFGBG-724841, ALFGBG-533131), the Swedish Society for Medical Research (SSMF), and Astra-Zeneca. Open access publication was funded by the University of Gothenburg. The funders had no influence on the study design, the collection, analysis and interpretation of data, the writing of the report, or the decision to submit the article for publication.

## CRediT authorship contribution statement

**Alina Skultecka:** Writing – review & editing, Writing – original draft, Methodology, Formal analysis, Conceptualization. **Fredrik Nyberg:** Writing – review & editing, Investigation, Funding acquisition, Conceptualization. **Lauren Lissner:** Writing – review & editing, Investigation, Funding acquisition, Conceptualization. **Maria Rosvall:** Writing – review & editing, Investigation, Funding acquisition, Conceptualization. **Dag S. Thelle:** Writing – review & editing, Investigation, Funding acquisition, Conceptualization. **Anna-Carin Olin:** Writing – review & editing, Investigation, Funding acquisition, Conceptualization. **Kjell Torén:** Writing – review & editing, Investigation, Funding acquisition, Conceptualization. **Lena Björck:** Writing – review & editing, Investigation, Funding acquisition, Conceptualization. **Annika Rosengren:** Writing – review & editing, Investigation, Funding acquisition, Conceptualization. **Kirsten Mehlig:** Writing – review & editing, Validation, Supervision, Methodology, Formal analysis, Data curation, Conceptualization.

## Declaration of competing interest

Anna-Carin Olin is one of the inventors of the PExA method and shareholder and board member of PExA AB. The authors declare no competing interests relevant to this manuscript.
