# Supplementary material for: Comparison of associations between alcohol consumption and metabolic syndrome according to three definitions: The Swedish INTERGENE study
Source: Metabol Open. 2024 Jun 5;23:100292. doi: 10.1016/j.metop.2024.100292 (PMC11231701; doi:10.1016/j.metop.2024.100292)
Supplement: Multimedia component 1 [file mmc1.docx]

**Fig S1:** Venn diagram for the prevalence of MetS according to three definitions (percentage among 773 participants with MetS according to at least one definition).


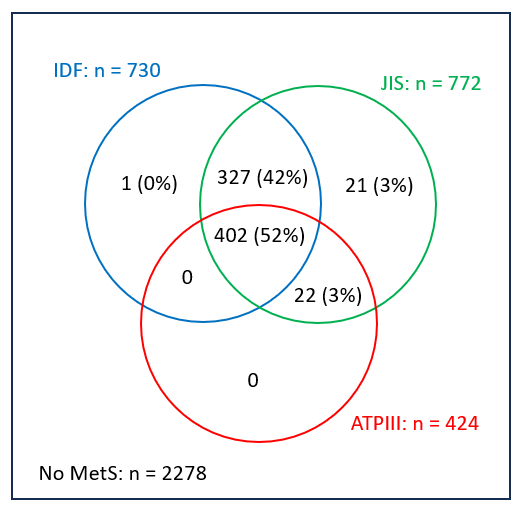


**Table S1:** Analysis of confounding of the association between metabolic syndrome according to the APTIII definition and its components and alcohol consumption (OR (95% CI) from model 2, total = 2912)

|  | Metabolic syndrome  (n = 400) | Central obesity  (n = 657) | Hypertension  (n = 1695) | Elevated glucose  (n = 214) | Elevated triglycerides  (n = 745) | | Low HDL cholesterol  (n = 300) | | |
| --- | --- | --- | --- | --- | --- | --- | --- | --- | --- |
| *Female sex* | 0.67 (0.53-0.84) * | 1.56 (1.28-1.91) *** | 0.60 (0.52-0.72) *** | 0.35 (0.25-0.48) *** | 0.43 (0.35-0.51) *** | | 0.86 (0.66-1.10) | | |
| *Nordic born* | 1.27 (0.84-1.90) | 1.75 (1.21-2.53) ** | 1.09 (0.79-1.48) | 1.30 (0.74-2.26) | 0.75 (0.56-1.02) | | 0.95 (0.64-1.43) | | |
| *Age (ref: 25-34 years)* |  |  |  |  |  | |  | | |
| 35-44 years | 1.77 (0.98-3.18) | 2.03 (1.29-3.20) ** | 2.33 (1.70-3.19) *** | 1.64 (0.63-4.30) | 1.60 (1.10-2.33) * | | 0.99 (0.65-1.53) | | |
| 45-54 years | 2.69 (1.51-4.77) *** | 2.76 (1.76-4.33) *** | 4.71 (3.42-6.49) *** | 3.48 (1.41-8.61) ** | 2.18 (1.50-3.17) *** | | 0.96 (0.62-1.51) | | |
| 55-64 years | 3.61 (2.06-6.35) *** | 3.46 (2.21-5.40) *** | 9.09 (6.53-12.6) *** | 6.87 (2.85-16.6) *** | 2.11 (1.45-3.07) *** | | 0.91 (0.58-1.42) | | |
| 65-77 years | 4.17 (2.21-7.85) *** | 4.07 (2.44-6.81) *** | 26.8 (16.7-43.0) *** | 7.52 (2.87-19.7) *** | 1.86 (1.18-2.93) ** | | 0.70 (0.40-1.24) | | |
| *Married/cohabitant* | 1.15 (0.87-1.52) | 1.09 (0.86-1.37) | 0.84 (0.68-1.05) | 0.82 (0.58-1.17) | 1.13 (0.91-1.41) | | 1.06 (0.79-1.42) | | |
| *Urban residence* | 0.96 (0.76-1.21) | 1.13 (0.93-1.37) | 0.58 (0.49-0.70) *** | 1.61 (1.19-2.18) ** | 0.97 (0.81, 1.17) | | 0.95 (0.74-1.23) | | |
| *Self-rated health (ref=very good)* | |  |  |  | |  | |  |  |
| Good | 1.90 (1.26-2.86)** | 2.03 (1.48-2.78) *** | 1.17 (0.94-1.46) | 1.42 (0.88-2.29) | 1.49 (1.14-1.93) ** | | 1.40 (0.96-2.06) | | |
| Mixed/poor | 3.46 (2.24-5.37)*** | 3.24 (2.29-4.59) *** | 1.56 (1.17-2.09) ** | 2.34 (1.38-4.00) ** | 1.95 (1.43-2.64) *** | | 1.51 (0.97-2.35) | | |
| *Heredity for diabetes* | 1.64 (1.25-2.16)*** | 1.62 (1.28-2.06) *** | 1.06 (0.83-1.34) | 2.00 (1.41-2.82) *** | 1.23 (0.97-1.55) | | 1.44 (1.06-1.97) * | | |
| *University education* | 0.79 (0.59-1.06) | 0.77 (0.61-0.98) * | 0.72 (0.59-0.87) *** | 0.92 (0.64-1.33) | 0.78 (0.63-0.97) * | | 0.87 (0.65-1.18) | | |
| *Low income* | 1.29 (0.95-1.76) | 1.35 (1.04-1.75) * | 0.89 (0.70-1.14) | 1.48 (0.98-2.23) | 1.11 (0.87-1.43) | | 1.47 (1.08-2.00) ** | | |
| *Working status (ref=working)* | |  |  |  | |  | |  |  |
| Retired | 1.27 (0.89-1.81) | 1.29 (0.95-1.74) | 1.15 (0.85-1.55) | 1.22 (0.77-1.94) | 1.33 (0.99-1.78) | | 1.47 (0.99-2.19) | | |
| Unemployed/other | 1.52 (0.99-2.33) | 1.28 (0.89-1.83) | 1.00 (0.73-1.37) | 1.10 (0.59-2.03) | 0.93 (0.65-1.33) | | 1.78 (1.19-2.66) ** | | |
| *Physical activity (ref=sedentary)* | |  |  |  | |  | |  |  |
| Moderate | 0.61 (0.44-0.86) ** | 0.55 (0.41-0.74) *** | 1.05 (0.78-1.42) | 0.91 (0.56-1.48) | 0.60 (0.46-0.79) *** | | 0.54 (0.38-0.78) *** | | |
| Regular training | 0.30 (0.19-0.47) *** | 0.30 (0.21-0.44) *** | 0.91 (0.66-1.27) | 0.62 (0.34-1.11) | 0.35 (0.25-0.48) *** | | 0.46 (0.30-0.70) *** | | |
| *Smoking (ref=never)* | |  |  |  | |  | |  |  |
| Former smoker | 1.35 (1.05-1.74) ** | 1.43 (1.15-1.77) ** | 0.99 (0.81-1.21) | 1.05 (0.76-1.45) | 1.07 (0.87-1.31) | | 0.93 (0.69-1.25) | | |
| Current smoker | 0.96 (0.69-1.35) | 1.09 (0.83-1.43) | 0.69 (0.54-0.88) ** | 0.47 (0.28-0.78) ** | 1.21 (0.94-1.57) | | 1.31 (0.93-1.83) | | |
| *Coffee consumption (ref=none)* | |  |  |  | |  | |  |  |
| < 3 cups/day | 0.84 (0.55, 1.31) | 1.55 (1.04, 2.32) * | 1.05 (0.76, 1.45) | 1.18 (0.63, 2.19) | 0.98 (0.70, 1.38) | | 0.79 (0.52, 1.20) | | |
| ≥ 3 cups/day | 0.83 (0.54, 1.27) | 1.40 (0.94, 2.09) | 0.98 (0.71, 1.34) | 0.90 (0.49, 1.68) | 0.93 (0.66, 1.30) | | 0.70 (0.46, 1.06) | | |
| AUROC | 0.76 | 0.75 | 0.80 | 0.79 | 0.70 | | 0.69 | | |

* p-value < 0.05, ** p-value < 0.01, *** p-value < 0.001

**Table S2:** Mean percentage (SD) of ethanol intake from beer, wine, and spirits overall and by category of total ethanol intake among consumers.

|  |  | Categories of total ethanol intake based on sex-specific tertiles of ethanol intake among consumers | | |
| --- | --- | --- | --- | --- |
| Subtype | Overall | Low | Medium | High |
| Beer | 39.9 (29.6) | 40.1 (35.5) | 39.3 (26.4) | 40.5 (26.0) |
| Wine | 46.3 (30.9) | 45.4 (37.0) | 46.6 (28.4) | 47.0 (26.5) |
| Spirits | 13.7 (17.1) | 14.5 (22.8) | 14.1 (15.0) | 12.5 (11.4) |

**Table S3:** Associations between ethanol intake from beer, wine, and spirits in mutually adjusted models for Metabolic Syndrome (MetS) and its component low HDLcholesterol, adjusted for age and sex, and fully adjusted.

|  |  |  | Ethanol intake (ref = low) | | |
| --- | --- | --- | --- | --- | --- |
|  |  |  | Abstinence | Medium | High |
| Outcome | Cases/total | Type | OR  (95% CI) | OR  (95% CI) | OR  (95% CI) |
| MetS – ATPIII, age-sex adjusted | 424/3051 | Beer | 1.24  (0.95, 1.63) | 0.80  (0.57, 1.12) | 0.66  (0.40, 1.09) |
|  |  | Wine | **1.52****  **(1.17, 1.97)** | 0.82  (0.59, 1.15) | **0.58***  **(0.34, 0.99)** |
|  |  | Spirits | 0.96  (0.75, 1.24) | 0.91  (0.35, 2.34) | 2.19  (0.42, 11.4) |
| MetS – ATPIII, fully adjusted ^a^ | 400/2912 | Beer | 1.31  (0.98, 1.75) | 0.82  (0.58, 1.17) | 0.60  (0.35, 1.02) |
|  |  | Wine | 1.20  (0.90, 1.60) | 0.88  (0.61, 1.27) | 0.71  (0.41, 1.24) |
|  |  | Spirits | 0.98  (0.75, 1.28) | 1.20  (0.46, 3.16) | 1.76  (0.31, 10.0) |
| Low HDL cholesterol, age-sex adjusted | 317/3051 | Beer | **1.37***  **(1.02, 1.83)** | 0.80  (0.53, 1.19) | 0.62  (0.33, 1.18) |
|  |  | Wine | 1.28  (0.96, 1.71) | **0.57****  **(0.39, 0.86)** | **0.46***  **(0.23, 0.93)** |
|  |  | Spirits | 1.14  (0.86, 1.50) | 0.24  (0.03, 1.72) | **5.52***  **(1.03, 29.6)** |
| Low HDL cholesterol, fully adjusted ^a^ | 300/2912 | Beer | 1.34  (0.98, 1.82) | 0.80  (0.53, 1.21) | 0.59  (0.30, 1.15) |
|  |  | Wine | 1.02  (0.75, 1.39) | **0.56***  **(0.37, 0.87)** | 0.56  (0.27, 1.13) |
|  |  | Spirits | 1.11  (0.83, 1.48) | 0.27  (0.04, 1.96) | 3.60  (0.61, 21.1) |

* p-value < 0.05, ** p-value < 0.01, *** p-value < 0.001

Metabolic Syndrome (MetS)

National Cholesterol Education Program Adult Treatment Panel III (ATP III)

^a^ Adjusted for sex, age, heredity for diabetes, born in Nordic countries, urban residence, married/cohabitant, self-rated health, education, income, working status, physical activity, smoking status, coffee consumption.
